# Discriminating between proposed Brain-First and Body-First Parkinson’s disease using conventional and radiomics-enhanced dopamine transporter SPECT image analysis

**DOI:** 10.1038/s41531-025-01164-z

**Published:** 2025-11-20

**Authors:** Giovanni Palermo, Gayanè Aghakhanyan, Gabriele Bellini, Sara Giannoni, Gabriele Vadi, Roberto Francischello, Giovanna Nonne, Maria Giulia Tedeschi, Riccardo Morganti, Daniela Frosini, Nicola Pavese, Duccio Volterrani, Roberto Ceravolo

**Affiliations:** 1 Center for Neurodegenerative Diseases—Parkinson’s Disease and Movement Disorders, Unit of Neurology, Department of Clinical and Experimental Medicine, University of Pisa, Pisa, Italy; 2 Neurology Unit, Department of Clinical and Experimental Medicine, University of Pisa, Pisa, Italy; 3 Regional Center of Nuclear Medicine, Department of Translational Research and of New Surgical and Medical Technology, University of Pisa, Pisa, Italy; 4 Neurology Department, San Giuseppe Hospital, Empoli, Italy; 5 Department of Translational Research and of New Surgical and Medical Technology, Academic Radiology, University of Pisa, Pisa, Italy; 6 Section of Statistics, University of Pisa, Pisa, Italy; 7 Clinical Ageing Research Unit, Translational and Clinical Research Institute, Newcastle University, Newcastle upon Tyne, UK; 8 Department of Clinical Medicine, Nuclear Medicine and PET, Aarhus University, Aarhus, Denmark

**Keywords:** Neurology, Diagnostic markers

## Abstract

Two Parkinson’s disease subtypes—“Brain-First” and “Body-First”—have been proposed based on putative sites of onset. We examined whether “Body-First” markers relate to more symmetric striatal [^123^I]-FP-CIT uptake and whether imaging could discriminate the subtypes. In a retrospective cohort of 158 de novo PD patients imaged at diagnosis and followed for six years, patients were classified as “Body-First” if baseline REM sleep behavior disorder, constipation, or neurogenic orthostatic hypotension was present. DaTQUANT provided semiquantitative metrics; a radiomics-based classifier was also trained on DAT-SPECT images. Neither asymmetry indices nor other DaTQUANT measures differed between groups (all *p* > 0.05). Radiomics showed poor discrimination (AUC ≈ 0.46). Clinically, “Body-First” patients displayed a more adverse course, with higher MCI prevalence and greater MMSE decline and neuropsychiatric burden, whereas motor severity and complications were comparable between groups. These data suggest DAT-SPECT—conventional or radiomics-enhanced—does not separate proposed subtypes at diagnosis, although “Body-First” features forecast worse non-motor progression.

## Introduction

Parkinson’s disease (PD) is a common neurodegenerative disorder primarily characterized by bradykinesia, rigidity, and resting tremor, which are largely attributed to the loss of dopaminergic neurons. However, PD is increasingly recognized as a highly heterogeneous condition, encompassing a broad spectrum of non-motor symptoms linked to dysfunction across multiple brain regions and neurotransmitter pathways^1^. Key non-motor symptoms—such as REM sleep behavior disorder (RBD), olfactory loss, autonomic dysfunction, anxiety, depression, and constipation—are well-established prodromal markers of the disease^2^. The diverse clinical presentations, varying rates and patterns of progression, and neuropathological variability suggest the existence of biologically distinct subtypes^3^.

Early attempts at subtyping PD focused on clustering based on demographic factors (e.g., age at onset) or clinical features (e.g., motor phenotype)^4^. Recently, a different subtype classification system has been proposed, based on the temporal appearance and propagation of α-synuclein (α-syn), termed the α-syn Origin site and Connectome-SOC^5,6^.

In this binary classification system, the “Body-First” phenotype is characterized by pathological α-syn aggregates originating in the enteric nervous system, which then spread to the central nervous system via autonomic connections. In contrast, the “Brain-First” phenotype involves initial damage to higher Braak stage structures, such as the substantia nigra, before Lewy pathology extends to peripheral regions^5^. Notably, patients with the “Body-First” phenotype typically experience a prolonged prodromal phase, marked by premotor RBD, a more aggressive clinical course, and ascending, symmetrical neurodegeneration. In these individuals, α-syn is hypothesized to propagate rostrally and bilaterally toward the brainstem via the vagus nerve and the synaptic connectome, ultimately leading to symmetrical nigrostriatal dysfunction^7^. This progression can be confirmed in vivo through brain imaging using 123I-*N*-ω-fluoropropyl-2β-carbomethoxy-3β-{4-iodophenyl}nortropane (^123^I-FP-CIT) single-photon emission computed tomography (SPECT), which measures dopamine transporter (DAT) activity.

Additional clinical markers for the “Body-First” subtype include constipation and neurogenic orthostatic hypotension (nOH)^6,8^. Both nOH and constipation have been strongly linked to RBD, reinforcing the idea of a “malignant” PD phenotype characterized by early cognitive decline and postural instability^9,10^. As such, early-stage PD patients with RBD are expected to show a higher incidence of constipation and nOH, along with greater symmetry in dopaminergic imaging, when compared to those without RBD. However, no specific marker currently exists to distinctly differentiate between these two primary PD subtypes. Additionally, studies investigating the nigrostriatal dopaminergic system in relation to the “Brain-First” vs “Body-First” dichotomy have yielded inconsistent results^11–13^.

Identifying distinct patient subgroups is crucial for advancing our understanding of the underlying pathophysiology, which could have significant implications for disease progression, prognosis, and therapeutic strategies.

The primary aim of our study was to compare the clinical characteristics and dopamine transporter imaging patterns of possible “Body-First” and “Brain-First” de novo PD patients. Patients were classified based on the presence of “Body-First” phenotype clinical markers, including RBD, nOH, and constipation. To further differentiate between the subtypes, we employed a radiomics-enhanced analysis of DAT-SPECT images for automated classification. The secondary objective was to evaluate the long-term clinical progression of these subtypes. Patients were retrospectively followed for a minimum of six years, allowing us to assess the long-term impact of the baseline classification at the final follow-up.

## Results

### Baseline clinical evaluation

A total of 158 early de novo PD participants were included in this study. The baseline (T0) demographic and clinical characteristics are summarized in Tables 1 and 2. Fourteen subjects (8.9%) fulfilled the diagnosis of PD-MCI.

**Table 1:** Demographic and clinical features of PD patients included at baseline

Number	158
Age at inclusion, y	64.2 ± 10.1
Age at symptom onset, y	62.9 ± 10
Female/male, *n*. (%)	68/90 (43/57)
Disease duration (months)	13.3 ± 9.7
Predominant side of motor symptoms	
Right, *n*. (%)	73 (46.5)
Left, *n*. (%)	78 (49.7)
Bilateral, *n*. (%)	6 (3.8)
MDS-UPDRS motor subscore (off state)	18.9 ± 7.6
MMSE	29.2 ± 1.5

Values are mean ± standard deviation (SD) if not otherwise indicated.

*MDS-UPDRS* MDS-sponsored revision of the unified Parkinson’s disease rating scale, *MMSE* mini-mental state examination.

**Table 2:** Frequency of nonmotor symptoms in patients with PD at baseline

RBD, *n*. (%)	40 (25.3)
Constipation, *n*. (%)	21 (13.3)
nOH, *n*. (%)	7 (4.4)
RBDSQ (±s.d.)	5.3 ± 2.0
SCOPA-AUT Urinary domain (total score ≥ 1), *n*. (%)	21 (13.3)
HAM-D (total score ≥ 13), *n*. (%)	55 (36.7)
HAM-A (total score ≥10), *n*. (%)	39 (25.3)
MDS-UPDRS 1.1 (score = 1), *n*. (%)	22 (13.9)
MCI, *n*. (%)	14 (8.9)

*HAM-A* Hamilton anxiety scale, *HAM-D* Hamilton depression scale, *MCI* mild cognitive impairment, *MDS-UPDRS* MDS-sponsored revision of the unified Parkinson’s disease rating scale, *nOH* neurogenic orthostatic hypotension, *RBD* REM sleep behavior disorder, *RBDSQ* RBD screening questionnaire, *SCOPA-AUT* scale for outcomes in Parkinson’s disease for autonomic symptoms.

Patients were categorized based on the presence of RBD, constipation, and nOH as markers of the Body-First type. However, due to the small number of patients with baseline nOH (only 7, 4.4%) and the limited presence of premotor nOH symptoms reported during the interviews, we did not include a comparison between nOH^+^ and nOH^−^ groups in our analysis. Furthermore, our strict diagnostic criteria indicated that RBD, constipation, and nOH coexisted in only one patient at baseline, so we did not analyze patients based on the concurrent presence of all three features.

**Fig. 1:**
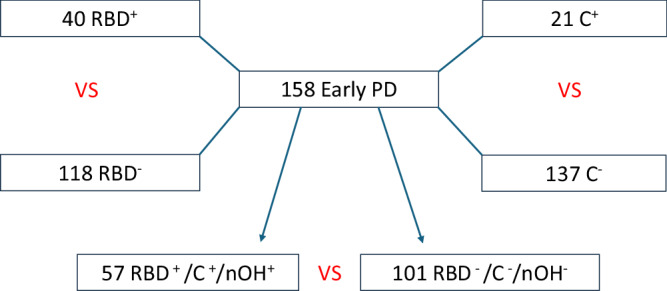
Cohort selection and comparison diagram. C^+^ patients with constipation, nOH^+^ patients with neurogenic orthostatic hypotension, PD Parkinson’s disease, RBD^+^ patients with REM sleep behavior disorder, RBD^+^/C^+^/nOH^+^ patients with at least one of RBD, constipation, or neurogenic orthostatic hypotension, RBD^–^/C^–^/nOH^–^ patients without any of RBD, constipation, or neurogenic orthostatic hypotension.

Instead, we created a group consisting of patients with at least one of these clinical features (RBD^+^/C^+^/nOH^+^) and compared them to those without any of these features (RBD^−^/C^−^/nOH^−^). Among the total cohort of 158 patients, 57 individuals were identified as having at least one of RBD, constipation, or nOH. Specifically, 40 patients were diagnosed with RBD, 21 with constipation, and 7 with nOH (Fig. 1). Notably, several patients presented more than one of these conditions, confirming a substantial overlap among them.

#### RBD^+^ vs RBD ^–^

The dataset included 40 de novo PD patients (25.3%) with premotor RBD (RBD^+^) and 118 de novo PD patients (74.6%) without premotor RBD (RBD^−^). There were no significant differences at baseline between the RBD^+^ and RBD− groups in terms of sex, age at onset, lateralization of motor symptoms, MDS-UPDRS-III scores, cognitive functioning, duration of Parkinsonism, cardiovascular and gastrointestinal symptoms on the SCOPA-AUT scale, or the frequency of depression and anxiety (all *p* > 0.05). As expected, RBD^+^ patients had significantly higher RBDSQ scores compared to RBD^−^ patients (8.9 ± 1.7 vs 2.9 ± 1.4, *p* < 0.001). A larger proportion of RBD^+^ patients also exhibited significant urinary symptoms on the SCOPA-AUT scale (22.5% vs 10.1%, *p* = 0.047). Detailed comparison of the two groups at baseline can be found in supplementary materials (Table S1).

#### C^+^ vs C^–^

Based on the presence of premotor constipation, we identified 21 de novo PD patients (13.3%) with constipation (C^+^) and 137 de novo PD patients (86.7%) without constipation (C^−^). Similar to the comparison between patients with and without RBD, there were no significant differences at baseline between the C^+^ and C^−^ groups regarding sex, lateralization of motor symptoms at onset, MDS-UPDRS-III scores, cognitive functioning, RBDSQ scores, duration of parkinsonism, cardiovascular and gastrointestinal symptoms on the SCOPA-AUT scale, or the frequency of depression and anxiety (*p* > 0.05). However, C^+^ patients were significantly older than C^−^ patients both at the time of assessment (68.4 ± 8.2 years vs 63.5 ± 10.2 years, *p* = 0.040) and at symptom onset (67.1 ± 8.2 years vs 62.3 ± 10.3 years, *p* = 0.046). Additionally, a significantly higher proportion of C^+^ patients experienced urinary symptoms compared to those without constipation (33.3% vs 10.2%, *p* = 0.004). Olfaction was also significantly reduced in the C^+^ group (47.6% vs 23.3%, *p* = 0.019). Detailed comparison of the two groups at baseline can be found in supplementary materials (Table S2).

#### RBD^+^/C^+^/nOH^+^ vs RBD^−^/C^−^/nOH^−^

Among patients with at least one premotor symptom—RBD, constipation, or nOH—the dataset included 57 de novo PD patients (36.1%) (RBD^+^/C^+^/nOH^+^), while 101 patients (63.9%) had none (RBD^−^/C^−^/nOH^−^). No significant differences were observed across most variables (*p* > 0.05) at baseline. However, subjective cognitive deficits were more prevalent in the RBD^+^/C^+^/nOH^+^ group (22.8% vs 8.9%, *p* = 0.015). Moreover, the group had a significantly higher prevalence of urinary symptoms (26.3% vs 5.9%, *p* < 0.001) compared to the RBD^−^/C^−^/nOH^−^ group, and there was a trend toward a higher prevalence of depressive symptoms (43.8% vs 29.7%, *p* = 0.073). Detailed comparison of the two groups at baseline is shown in Table 3.

**Table 3:** Comparison between RBD^+^/C^+^/nOH^+^ and RBD^−^/C^−^/nOH^−^ patients at baseline

	RBD^+^/C^+^/OH^+^ (n. 57)	RBD^−^/C^−^/OH^−^ (n. 101)	*p*-value
Female/male	23/34	45/56	0.608
Age at inclusion, y	65.9 ± 8.7	63.2 ± 10.7	0.104
Age at symptom onset, y	64.8 ± 8.7	61.9 ± 10.8	0.083
Disease duration (months)	13.6 ± 11.7	14.3 ± 13.7	0.720
Predominant side of motor symptoms (*n*, %)			0.438
Right	29 (50.8)	44 (43.6)	
Left	26 (45.6)	52 (51.5)	
Bilateral	2 (3.6)	5 (5)	
MDS-UPDRS motor subscore	19.7 ± 7.6	18.4 ± 7.5	0.250
MMSE	29.1 ± 1.2	29.2 ± 1.7	0.592
RBDSQ	6.0 ± 2.0	4.8 ± 1.8	**<0.001**
SCOPA-AUT urinary domain - (total score ≥ 1), *n*. (%)	15 (26.3)	6 (5.9)	**<0.001**
HAM-D (total score ≥ 13), *n*. (%)	25 (43.8)	30 (29.7)	0.073
HAM-A (total score ≥ 10), *n*. (%)	16 (28.1)	23 (22.7)	0.565
MDS-UPDRS 1.1 (score = 1), *n*. (%)	13 (22.8)	9 (8.9)	**0.015**
MCI, *n*. (%)	8 (14.0)	6 (5.9)	0.086

Values are mean ± SD if not otherwise indicated. Results in bold letters indicate significant difference (*p* < 0.05).

*HAM-*A Hamilton anxiety scale, *HAM-D* Hamilton depression scale, *MCI* mild cognitive impairment, *MDS-UPDRS* MDS-Sponsored Revision of the Unified Parkinson’s Disease Rating Scale, *RBD+/C+/nOH+* patients with at least one of RBD, constipation, or neurogenic orthostatic hypotension, *RBD−/C−/nOH−* patients without any of RBD, constipation, or neurogenic orthostatic hypotension, *RBDSQ* RBD screening questionnaire, *SCOPA-AUT* scale for outcomes in Parkinson’s disease for autonomic symptoms.

### DaTQUANT analysis results

The analysis of continuous variables from the DaTQUANT output, including SBRs, putamen-to-caudate ratios, asymmetry indices (AIs), and their Z-scores, showed no statistically significant differences between the RBD^+^/C^+^/nOH^+^ and RBD^−^/C^−^/nOH^−^ groups (Table 4). Similarly, no significant differences were found in the comparisons between the RBD^+^ and RBD^−^ groups or the C^+^ and C^−^ groups, with detailed results for these analyses provided in the supplementary materials (Table S3 and S4, respectively).

**Table 4:** DaTQUANT output for patients categorized by their RBD^−^/C^−^/nOH^−^ vs RBD^+^/C^+^/nOH^+^ status

Variables	RBD^+^/C^+^/nOH^+^ (n. 57)	RBD^−^/C^−^/nOH^−^ (n. 101)	*p*-value
Striatum right SBR	1.22 (0.38)	1.22 (0.34)	0.997
Striatum left SBR	1.44 (0.42)	1.43 (0.41)	0.875
Putamen right SBR	1.03 (0.31)	1.03 (0.30)	0.976
Putamen left SBR	1.22 (0.39)	1.20 (0.37)	0.807
Caudate right SBR	1.62 (0.56)	1.63 (0.55)	0.921
Caudate left SBR	1.91 (0.59)	1.90 (0.59)	0.938
Anterior putamen right SBR	1.20 (0.37)	1.19 (0.35)	0.966
Anterior putamen left SBR	1.42 (0.46)	1.38 (0.41)	0.604
Posterior putamen right SBR	0.68 (0.27)	0.68 (0.26)	0.973
Posterior putamen left SBR	0.81 (0.29)	0.84 (0.34)	0.507
Putamen to caudate right ratio	0.79 (0.10)	0.79 (0.14)	0.890
Putamen to caudate left ratio	0.77 (0.10)	0.78 (0.13)	0.927
Caudate asymmetry	0.13 (0.10)	0.12 (0.08)	0.640
Putamen asymmetry	0.11 (0.08)	0.12 (0.08)	0.473
Striatum asymmetry	0.12 (0.08)	0.11 (0.07)	0.956
*Z*-score striatum left SBR	−2.78 (0.86)	−2.96 (0.99)	0.261
*Z*-score striatum right SBR	−2.88 (1.14)	−3.09 (0.90)	0.264
*Z*-score caudate left SBR	−1.94 (1.03)	−2.11 (1.24)	0.366
*Z*-score caudate right SBR	−1.93 (1.41)	−2.11 (1.14)	0.430
*Z*-score putamen left SBR	−2.91 (0.91)	−3.14 (0.88)	0.148
*Z*-score putamen right SBR	−3.45 (0.81)	−3.63 (0.86)	0.211
*Z*-score anterior putamen left SBR	−2.60 (1.03)	−2.87 (0.90)	0.120
*Z*-score anterior putamen right SBR	−3.17 (0.87)	−3.34 (0.88)	0.271
*Z*-score posterior putamen left SBR	−3.27 (0.67)	−3.37 (0.91)	0.433
*Z*-score posterior putamen right SBR	−3.66 (0.76)	−3.86 (0.89)	0.161
*Z*-score striatum asymmetry	3.48 (3.36)	3.44 (3.14)	0.953
*Z*-score caudate asymmetry	2.33 (2.66)	2.09 (2.19)	0.589
*Z*-score putamen asymmetry	2.88 (3.15)	3.28 (2.95)	0.450
*Z*-score putamen to caudate left ratio	−2.30 (1.67)	−2.28 (2.03)	0.956
*Z*-score putamen to caudate right ratio	−2.15 (1.59)	−2.16 (2.18)	0.972

Data are presented as mean and SD.

*RBD+/C+/nOH+* patients with at least one of REM behavior disorder, constipation, or neurogenic orthostatic hypotension, *RBD–/C–/nOH–* patients without any of REM behavior disorder, constipation, or neurogenic orthostatic hypotension, *SBR* specific binding ratio.

#### Radiomics analysis

The radiomics analysis aimed at distinguishing between RBD^+^/C^+^/nOH^+^ and RBD^−^/C^−^/nOH^−^ groups demonstrated suboptimal model performance. The model achieved an area under the curve (AUC) of 0.46 ± 0.1, indicating limited efficacy in differentiating between these PD subtypes. Similarly, the classifier’s accuracy was 0.49 ± 0.1, reflecting modest performance in correctly classifying the subtypes. When applied to differentiate between RBD^+^ and RBD^−^ groups, as well as C^+^ and C^−^ groups, the model yielded comparable results, further highlighting its limited discriminative capability.

### Association between DaTQUANT SBRs and clinical variables

Multivariate multiple regression analysis across all models revealed that the “Brain-First” and “Body-First” subtypes, when considered as group variables, were not significantly associated with any of the SBR measures, putamen to caudate ratios, or AIs. Specifically, the *p*-values for these subtypes ranged from 0.3407 to 0.4747, indicating that, after adjusting for other covariates, they do not significantly influence SBR values in any of the analyzed regions. In contrast, when considering the entire population, other variables such as sex, age, and MDS-UPDRS-III scores were identified as significant predictors of SBR values in specific regions when considering the whole population. For instance, sex was significantly associated with SBR values in the left caudate (*p* = 0.000639), while age was a significant predictor in several regions, including the left striatum (*p* = 0.002184) and right caudate (*p* = 0.0066). Additionally, MDS-UPDRS motor scores were significant predictors of SBR in regions such as the right putamen (*p* = 0.035) and left putamen (*p* = 0.01271).

Separate multivariate regression models for the “Brain-First” subgroup revealed a similar pattern, with significant predictors of SBR values including sex, age at diagnosis, and MDS-UPDRS-III scores. Male sex was associated with lower SBR values in both the right striatum (β = −0.146, *p* = 0.038) and left striatum (β = −0.176, *p* = 0.034). Age at diagnosis showed a significant negative association with SBR values in the left striatum (β = −0.011, *p* = 0.006) and left caudate (β = −0.015, *p* = 0.007). MDS-UPDRS motor scores exhibited marginal significance in the right striatum (β = −0.009, *p* = 0.059).

In contrast, the “Body-First” subgroup exhibited fewer significant associations. Male sex was a significant predictor in only a few regions, such as the left putamen (β = −0.270, *p* = 0.015) and left caudate (β = −0.391, *p* = 0.028). Neither age at diagnosis nor MDS-UPDRS scores showed consistent associations with SBR values. These weaker associations suggest that dopaminergic deficits in the “Body-First” subtype are less strongly associated with clinical and demographic factors compared to the “Brain-First” subtype.

### Longitudinal clinical analysis

Longitudinal assessments in the overall cohort showed an increase in MDS-UPDRS-III scores from 18.87 ± 7.57 at baseline (T0) to 29.72 ± 12.76 at the last assessment (T1). LEDD values also increased from 328.9 ± 147.0 at the two-year assessment to 588.6 ± 263.1 at T1. At T1, 57 (36%) patients developed motor fluctuations, while 65 (41%) experienced dyskinesias. When comparing the previously identified “Body-First” and “Brain-First” subtypes, as well as the RBD^+^ and RBD^−^ and C+ and C^−^ groups (Table 5), no significant differences were found in motor scores progression, the longitudinal increase in dopaminergic dosage, or the development of motor complications. To account for the potential collinearity between dopaminergic treatment and motor severity, these comparisons were conducted using ANCOVA models, adjusting LEDD and MDS-UPDRS-III for each other.

**Table 5:** Percent change in MDS-UPDRS motor subscores and MMSE from baseline (T0) to last assessment (T1) across subgroups

	UPDRS T0	UPDRS T1	Δ%	*p*	MMSE T0	MMSE T1	Δ%	*p*
RBD^+^	19.5 ± 7.6	30.8 ± 13.6	+82.3 ± 92.4	0.822	29.1 ± 1.3	26.1 ± 5.7	−10.4 ± 18.5	**0.047**
RBD^−^	18.7 ± 7.6	29.3 ± 12.5	+78.0 ± 94.8		29.2 ± 1.6	28.0 ± 3.9	−3.7 ± 11.6	
C^+^	20.1 ± ± 7.5	32.1 ± 10.4	+76.5 ± 75.5	0.894	28.9 ± 1.2	27.0 ± 5.2	−7.4 ± 16.8	0.540
C^−^	18.7 ± 7.6	29.3 ± 13.1	+79.6 ± 97.0		29.2 ± 1.6	27.6 ± 4.4	−5.1 ± 13.5	
RBD^+^/C^+^/nOH^+^	19.7 ± 7.6	31.25 ± 13.13	+79.3 ± 86.0	0.989	29.1 ± 1.2	26.5 ± 5.0	−8.6 ± 16.1	**0.015**
RBD^−^/C^−^/nOH^−^	18.4 ± 7.5	28.80 ± 12.52	+79.0 ± 98.8		29.2 ± 1.7	28.1 ± 4.1	−3.6 ± 12.2	

Data are presented as mean ± SD. Results in bold letters indicate significant difference (*p* < 0.05).

*C+* patients with constipation, *MMSE* mini-mental state examination, *MDS-UPDRS* MDS-sponsored revision of the unified Parkinson’s disease rating scale, *RBD+* patients with REM sleep behavior disorder, *RBD+/C+/nOH+* patients with at least one of RBD, constipation, or neurogenic orthostatic hypotension, *RBD−/C−/nOH−* patients without any of RBD, constipation, or neurogenic orthostatic hypotension.

**Fig. 2:**
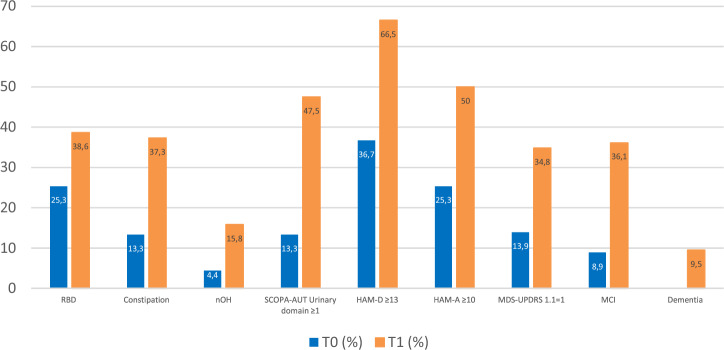
Prevalence of non-motor symptoms across the entire cohort at baseline (T0) and at the last assessment (T1). HAM-A Hamilton anxiety scale, HAM-D Hamilton depression scale, MCI mild cognitive impairment, MDS-UPDRS MDS-sponsored revision of the unified Parkinson’s disease rating scale, nOH neurogenic orthostatic hypotension, SCOPA-AUT scale for outcomes in Parkinson’s disease for autonomic symptoms.

Concerning non-motor symptoms, all showed an overall increase in the entire cohort by the last assessment (T1) (Fig. 2). MMSE scores declined from 29.2 ± 1.5 at baseline to 27.6 ± 4.5 at T1. The most notable increases over a minimum six-year follow-up period were observed in urinary symptoms (13.3% at T0 vs 47.5% at T1) and psychiatric disorders, with anxiety rising from 25.3% at T0 to 50% at T1 and depression from 36.7% at T0 to 66.5% at T1. Notably, cognitive decline varied significantly between groups (Table 5), with a greater percentage reduction in MMSE scores in RBD^+^/C^+^/nOH^+^ patients compared to RBD^−^/C^−^/nOH^−^ patients (*p* = 0.015) and in the RBD^+^ group compared to the RBD^−^ group (*p* = 0.047). Additionally, the number of patients diagnosed with MCI (but not dementia) was significantly higher in all subgroups (RBD^+^/C^+^/nOH^+^ vs RBD^−^/C^−^/nOH^−^, *p* < 0.001; RBD^+^ vs RBD^−^, *p* = 0.004; C^+^ vs C^−^, *p* = 0.031). Psychiatric symptoms (*p* = 0.019) were also more prevalent in RBD^+^/C^+^/nOH+ patients compared to the RBD^−^/C^−^/nOH^−^ group (Table 6). Further details on the RBD^+^ vs RBD^−^ and C^+^ vs C^−^ comparisons at T1 can be found in the supplementary materials (Tables S5 and S6). Additional data on the prevalence and severity of motor and non-motor symptoms at the last follow-up are also available (Table S7).

**Table 6:** Comparison between RBD^+^/C^+^/nOH^+^ and RBD^−^/C^−^/nOH^−^ patients at T1

	RBD^+^/C^+^/nOH^+^ (n. 57)	RBD^−^/C^−^/nOH^−^ (n. 101)	*p*-value
Age	72.1 ± 8.7	70.2 ± 10.1	0.237
MDS-UPDRS motor subscore	31.5 ± 13.1	28.8 ± 12.5	0.331^*^
LEDD	574.9 ± 232.5	596.6 ± 280.3	0.691^*^
MMSE	26.5 ± 5.0	28.1 ± 4.1	**0.032**
RBDSQ	6.3 ± 2.1	5.0 ± 1.8	**<0.001**
SCOPA-AUT urinary domain—(total score ≥ 1), *n*. (%)	41 (71.9)	54 (53.4)	**0.022**
HAM-D (total score ≥ 13), *n*. (%)	45 (78.9)	60 (59.4)	**0.012**
HAM-A (total score ≥ 10), *n*. (%)	35 (61.4)	44 (43.5)	**0.007**
MDS-UPDRS 1.1 (score = 1), *n*. (%)	27 (47.4)	28 (27.7)	**0.013**
MCI, *n*. (%)	31 (54.4)	26 (25.7)	**<0.001**
Dementia, *n*. (%)	8 (14.0)	7 (6.9)	0.143

Values are mean ± SD if not otherwise indicated. Results in bold letters indicate significant difference (*p* < 0.05).

*HAM-A* Hamilton anxiety scale, *HAM-D* Hamilton depression scale, *LEDD* Levodopa equivalent dose, *MCI* mild cognitive impairment, *MDS-UPDRS* MDS-sponsored revision of the unified Parkinson’s disease rating scale, *RBD+/C+/nOH+* patients with at least one of RBD, constipation, or neurogenic orthostatic hypotension, *RBD−/C−/nOH−* patients without any of RBD, constipation, or neurogenic orthostatic hypotension, *RBDSQ* RBD screening questionnaire, *SCOPA-AUT* scale for outcomes in Parkinson’s disease for autonomic symptoms.

*p-values for LEDD and MDS-UPDRS motor subscore were obtained via ANCOVA adjusted for MDS-UPDRS and LEDD, respectively.

## Discussion

Our findings suggest that dopaminergic imaging, whether conventional or radiomic, does not effectively differentiate between the “Body-First” and “Brain-First” clinical phenotypes in a de novo PD cohort characterized by distinct features of the “Body-First” phenotype. Patients were classified a priori based on the presence or absence of key clinical markers associated with the “Body-First” subtype, including RBD, nOH, and constipation. This approach also enabled us to assess concordance of these markers both at baseline and during follow-up, recognizing that not all patients exhibit all three features.

Regardless of the clinical marker used to distinguish between the “Body-First” and “Brain-First” subtypes, no differences were observed in the extent of nigrostriatal denervation or the degree of asymmetry in dopaminergic binding between the two groups. Clinically, however, the presence of non-motor symptoms at onset—notably RBD, constipation, and nOH—appears to correlate with a more ‘malignant’ phenotype, particularly in terms of disease progression, although these symptoms do not consistently cluster in the same individuals. This is evidenced by the tendency for patients with RBD, constipation, or nOH at onset (especially those with at least one of these non-motor symptoms) to develop more pronounced neuropsychiatric symptoms, urinary disturbances, and cognitive decline over six years of disease progression.

The absence of differentiable outcomes in our radiomics and DaTQUANT analyses may be attributed to the timing of imaging relative to disease progression. According to the SOC model of PD pathogenesis^5,6^, differences between “Brain-First” and “Body-First” PD subtypes are most evident in the early stages of the disease. This model suggests that α-syn pathology originates at specific sites and spreads through the brain’s connectome, leading to neurodegeneration. As the disease progresses, the pathology becomes more widespread, causing the clinical and imaging profiles of the two subtypes to converge. Thus, our findings may reflect a later stage of the disease, when these differences have diminished or become indistinguishable. This interpretation is consistent with observations from related α-synucleinopathies. In particular, previous imaging studies have shown that dementia with Lewy bodies (DLB)—considered predominantly a “Body-First” condition in the SOC model—exhibits more symmetric striatal dopaminergic degeneration than PD. Fedorova et al.^14^ reported lower putamen and caudate asymmetry using [^18^F]-FE-PE2I PET in DLB compared to PD patients, and Walker and colleagues^15^ similarly found more uniform [^123^I]FP-CIT binding within the posterior putamina in DLB patients compared to PD subjects. These findings reinforce the SOC model prediction and provide a useful reference point when interpreting asymmetry patterns in PD subtypes.

Earlier studies in de novo or early-stage PD patients with RBD have reported more pronounced DAT loss compared to those without RBD^16,17^. Arnaldi et al.^18^ further identified a specific pattern of nigro-caudate deafferentation in such patients, suggesting a distinct neurodegenerative process linking RBD and PD. More recently, Cicero and colleagues^19^ found that patients whose RBD preceded motor onset had lower caudate binding compared with “Brain-First” cases. In contrast, other studies support our findings, reporting no differences in DAT levels between PD patients with and without RBD^20,21^.

There are also conflicting findings regarding the association between RBD -considered the most reliable indicator of the “Body-First phenotype”- and the symmetry of striatal DAT binding in PD patients at diagnosis. Some studies, including Woo et al.^22^ and others^19,23^ have reported more symmetric nigrostriatal denervation in RBD-positive cases, whereas others, including our own, have failed to replicate this relationship^13,24^. This inconsistency likely reflects the complexity of the phenomenon, which may represent a group-level trend rather than a binary distinction. Discrepancies across studies may arise from differences in patient selection (e.g., inclusion of prodromal vs de novo PD), diagnostic approach for RBD (v-PSG confirmation vs questionnaires), criteria for other symptoms such as constipation or orthostatic hypotension, the operational definition of “premotor” RBD, disease stage at the time of imaging, and the proportion of patients meeting strict “Body-First” criteria. Differences in imaging modality and resolution (SPECT vs PET) may also play a role. In particular, symmetry differences may be most evident in the prodromal or very early motor phases, when degeneration patterns in “Brain-First” and “Body-First” phenotypes are still divergent, but tend to converge as the disease progresses.

The lack of differences in AIs of striatal DAT binding between the “Body-First” and “Brain-First” subtypes, when using constipation as a proxy for the “Body-First” phenotype, is consistent with another study that found no association between gastrointestinal disturbances and a more symmetrical dopaminergic binding in PD patients^12^. Similarly, in line with our findings, other reports have found no differences in the severity or asymmetry of dopaminergic denervation between constipated and non-constipated PD patients, despite a worse clinical phenotype in those with both constipation and RBD^25^. However, some studies have shown that constipation is significantly associated with declining striatal DAT availability, particularly in the caudate, in early PD^26^.

Similarly, while some studies have reported significantly lower ¹²³I-FP-CIT uptake in the putamen of PD patients with orthostatic hypotension compared to those without^27^, these findings have not been consistently replicated in other studies^28,29^.

Overall, our findings challenge the hypothesis that dopaminergic asymmetry could serve as a baseline marker to distinguish PD subtypes at onset. We observed a comparable pattern of dopaminergic dysfunction across groups, which were variably classified as clinical “Brain-First” and “Body-First “subtypes according to the SOC model. Additionally, we found that motor presentation was asymmetric in the vast majority of patients (>90%), regardless of their clinical classification into either subtype.

From a clinical perspective, and consistent with findings in the literature^30–32^, our results suggest a trend toward an association between RBD, constipation, and nOH, although the latter was observed in a limited number of newly diagnosed PD patients. This low prevalence of nOH in early PD aligns with a recent study by Baschieri et al.^33^, and prevented us from further investigating its independent contribution both at baseline and after the follow-up of six years in our cohort.

Conversely, the inclusion of constipation in our study was driven by the aim of maximizing the identification of patients with a potential “Body-First” phenotype. Constipation, an expression of autonomic dysfunction in PD^34^, is one of the earliest manifestations of the “Body-First” phenotype according to the SOC model^5,6^. This also provided an opportunity to explore whether patients labeled as “Body-First” based on the presence of constipation had a distinct clinical and DAT-SPECT profile compared to those without constipation.

The relationship between constipation and PD subtypes, as well as its impact on disease progression, has received less attention, but recent studies highlight that the severity of constipation at PD onset is correlated with faster motor and cognitive decline^35,36^.

In contrast, it is well-established that RBD, along with OH and MCI, is associated with an aggressive PD phenotype, characterized by the most rapid and malignant progression across both motor and non-motor components^32^. In line with these findings, our longitudinal data analysis reveals that both RBD and autonomic features are associated with the development of cognitive decline and neuropsychiatric manifestations over time, features that further support the “Body-First” subtype^6^.

Although our study does not aim to explore whether RBD and autonomic dysfunction can support or refute the clinical dichotomy of “Body-First”/“Brain-First” in patients with incident PD, our findings reaffirm the negative prognostic value of these non-motor clinical features^32^. Specifically, when present in various combinations at onset, these features appear to increase the risk of cognitive decline and the development of neuropsychiatric symptoms over six years of follow-up.

This could suggest that patients with RBD and autonomic dysfunction represent a more aggressive and widespread disease process. However, given that both RBD and autonomic dysfunction can precede the onset of motor symptoms in PD by many years, it becomes challenging to consider them part of a “malignant” disease spectrum, which is typically associated with more rapid progression.

Nonetheless, studies have reported that PD patients with RBD exhibit a greater range and density of α-syn deposition in the brain^37^, which pathologically supports the characterization of a malignant phenotype in these subtypes. It is important to note, however, that RBD is strongly associated with α-syn pathology, and the observed greater range and density of α-syn deposition in patients with RBD may reflect this inherent association rather than an independent marker of disease severity^38^.

On the other hand, in support of the “Body-First” etiology, the association between autonomic disturbances and RBD has been linked to a predominant peripheral deposition of α-syn, as evidenced by a higher load of deposits in colon biopsies and skin samples from patients with these clinical manifestations^39,40^. This is paralleled by a significantly greater degree of sympathetic cardiac denervation and colonic parasympathetic denervation in these patients compared to PD patients without RBD and autonomic dysfunction^41,42^.

We cannot determine whether the patients with RBD, constipation, or nOH included in our study have more widespread Lewy body pathology, particularly at the peripheral level, nor can we demonstrate a longer prodromal phase in these patients. However, this is highly likely, given the substantial body of evidence in the literature supporting this hypothesis^43^.

Nevertheless, the underlying mechanisms are likely more complex, with additional factors—such as inflammation, genetics, oxidative stress, mitochondrial dysfunction, and comorbidities—potentially playing significant roles in shaping the varied progression of PD^7^. Among these factors, the detection of asymmetry, one of the great mysteries of PD, may also be influenced by hemispheric dominance and handedness, which we did not specifically investigate in this study^44^.

Our findings suggest that the model of symmetric, bilateral involvement of the dopaminergic pathway in newly diagnosed PD patients with RBD and autonomic dysfunction cannot be fully supported. However, this does not entirely negate the “Body-First”/“Brain-First” dichotomy, which, in fact, seems to be indirectly supported by our clinical findings and multivariate multiple regression analysis. Notably, we observed that “Brain-First” PD patients exhibited stronger and more consistent associations between clinical variables—such as sex, age at diagnosis, and MDS-UPDRS motor scores—and dopaminergic dysfunction, reinforcing the idea that central mechanisms play a more prominent role in this subtype. In contrast, the “Body-First” subtype showed weaker and less consistent correlations, which aligns with the hypothesis that a predominant peripheral disease process, including autonomic dysfunction and non-central pathologies, underlies the progression of “Body-First” PD. Thus, it seems likely that newly diagnosed PD patients are no longer able to exhibit differences in DAT binding. As noted, the observation of greater asymmetry in idiopathic RBD patients is a consistent finding in the literature^11,13^, suggesting that the validity of the “Brain-First”/“Body-First” model based on dopaminergic asymmetry may only be demonstrable before the motor diagnosis of PD. Whether or not the “Brain-First”/“Body-First” hypothesis is taken into account, the partial lack of baseline differences in motor and non-motor profiles between patients with RBD/autonomic dysfunction and those without is not unexpected.

Numerous studies have reported similar findings, emphasizing the strong prognostic significance of these features in predicting long-term clinical outcomes^21,32^. Notably, a trend toward increased cognitive and psychiatric disturbances was already evident at baseline, as patients with at least one non-motor feature of the “Body-First” subtype tend to experience more cognitive and psychiatric disturbances. While this association is noticeable from the outset, it becomes more pronounced as the disease progresses.

Moreover, even the presence of a single feature—such as RBD, constipation, or nOH—appears to be an independent marker of disease severity, primarily influencing cognitive and psychiatric deterioration rather than motor progression. Lastly, the association of clinical markers of the “Body-First” type PD with urinary symptoms is both intriguing and perhaps unsurprising, as these disturbances also reflect underlying autonomic nervous system dysfunction. Frequently observed even in the early stages of untreated PD^45^, urinary symptoms have been variably linked to greater disease severity and reduced quality of life^46,47^. However, studies specifically exploring the independent contribution of early urinary disturbances to a worse disease prognosis remain limited. Considering that more severe urinary dysfunctions are typically expected in atypical Parkinsonism (especially multiple system atrophy) compared to PD, within the clinical continuum of alpha-synucleinopathies, the presence of early urinary symptoms in PD appears to be implicitly associated with a poorer prognosis. It is important to note that, as an exploratory study, we did not account for the numerous conditions that may also influence continence and bladder emptying. Furthermore, information regarding the nature of the observed urinary symptoms—whether irritative or obstructive—was not derived, which limits the scope of interpretation.

Another important limitation of this study is the inherent challenge of correctly classifying patients based on the presence of premotor symptoms, such as RBD, constipation, or neurogenic orthostatic hypotension. Although we made efforts to document these symptoms at baseline (looking for an objective confirmation for RBD through video-polysomnography and for nOH through blood pressure and heart rate measurements), and retrospectively confirm their presence during the prodromal phase, misclassification cannot be completely ruled out. Also, we cannot exclude the possibility that the percentage of patients with polysomnographically confirmed RBD may be higher than observed, as polysomnography was only performed in those who exhibited symptoms suggestive of RBD at baseline or in the period preceding diagnosis. These false negatives could potentially mask differences between the two groups, including in scintigraphic indices. In addition, the precise timing of RBD onset could not be reliably documented, preventing an analysis of RBD occurring before vs after the onset of Parkinsonism.

Premotor RBD alone, as well as constipation and nOH^−^, should not be considered definitive criteria for distinguishing “Brain-First” from “Body-First” PD subtypes, as highlighted in recent literature^48^. This complexity underscores the need for further longitudinal studies to refine these classifications. In our study, we deliberately adopted an inclusive approach by grouping patients with at least one among RBD, constipation, or nOH to maximize sensitivity in identifying possible “Body-First” features. We acknowledge that this broader definition may reduce specificity, particularly when features like constipation or nOH are considered in isolation. However, analyses restricted to RBD+ vs RBD– alone—one of the most robust clinical markers of the “Body-First” subtype—yielded comparable results in terms of imaging and clinical outcomes, suggesting that our main conclusions remain valid regardless of the grouping strategy. Nevertheless, future studies may benefit from using RBD alone—or in combination with objective biomarkers— as stricter criteria for body-first classification, particularly in prospective settings. Among these, ^123^I-meta-iodobenzylguanidine (MIBG) represents a promising surrogate marker of peripheral sympathetic denervation in PD^23^ and may help improve the specificity of body-first subtype identification, especially in de novo cohorts. Objective olfactory testing, such as UPSIT, also represents a potentially valuable tool in this context. However, neither MIBG scintigraphy nor UPSIT was performed in the present study, and their absence represents a minor methodological limitation.

More broadly, it should be noted that this study was a retrospective analysis of an existing dataset that had not been specifically designed to investigate the “Brain-First”/“Body-First” hypothesis. While we applied predefined classification criteria and attempted to exclude ambiguous cases, prospective studies with hypothesis-driven designs and clearly defined inclusion and exclusion rules will be necessary to validate and extend our findings.

The prevalence of constipation in our sample was lower than in several previous reports, probably due to the use of a strict double-definition combining SCOPA-AUT frequency thresholds with the Rome IV criteria. While this approach increases specificity, it may reduce sensitivity, thereby excluding milder cases and underestimating the overall prevalence. This methodological choice may have also limited the impact of constipation on our classification model, especially considering its relatively low discriminative power compared to RBD. Moreover, comparing subjective constipation prevalence in our PD population with that reported in the literature is challenging, as it varies widely across studies (from 8% to 70%), depending on the specific definitions of constipation used^49^.

In contrast, our RBD prevalence—approximately 25%—appears consistent with that reported in the largest cohort of *de-novo* PD patients examined using vPSG^50^. However, it should be emphasized that our study was not designed to estimate the prevalence of non-motor symptoms. Patients were recruited from a consecutive clinical sample, and a substantial number declined participation. Furthermore, we assessed numerous clinical and imaging outcomes, and as this was an exploratory analysis, no corrections were applied for multiple comparisons. Therefore, some of our positive findings may be due to chance.

Regarding the demographic characteristics of the RBD subgroups, we did not observe a significant age difference between patients with RBD^+^ and RBD^–^, in contrast to several previous reports. This discrepancy may reflect methodological factors, including whether RBD was identified solely via questionnaires or confirmed with v-PSG. The relatively modest number of v-PSG-confirmed RBD cases may have also limited the statistical power to detect small age differences. Additionally, the restriction to de novo, early-stage PD patients likely contributed to a more homogeneous age distribution, a finding also reported in other studies using similar cohorts^51^.

A further methodological consideration relates to the 36-month cutoff from motor symptom onset adopted for patient inclusion. While this window may appear broader than that used in some other studies, it was chosen pragmatically to reflect the real-world referral patterns of our center and to ensure adequate sample size for longitudinal analysis. All patients were still in early-stage PD at baseline, based on both clinical and imaging criteria. However, narrower inclusion windows may be preferable in future prospective studies focused on prodromal or ultra-early disease phases.

Additional limitations include the lack of a pathological confirmation and the single-center nature of the study. However, this study has several strengths, including a standardized method for PD diagnosis and evaluation, and the large number of subjects included. The larger size of our sample enhances the robustness and generalizability of our findings, especially in comparison to earlier studies that relied on smaller cohorts, some of which combined idiopathic RBD patients with those diagnosed with premotor RBD-PD to increase sample size^11^. All RBD patients had a diagnosis of RBD confirmed by polysomnography, and the clinical assessment of nOH in the study did allow for the confirmation or exclusion of its neurogenic origin in all patients. One of the key strengths is that it includes de novo PD patients, as dopaminergic therapy can influence non-motor symptoms, including cognition and mood, which might otherwise confound the results. Additionally, the relatively long follow-up period, with patients being consistently monitored since their diagnosis through regular annual evaluations over a 6-year period, offers valuable insight into the real-world progression of PD in relation to baseline features. It is important to note, however, that the primary aim of the study was not to explore the evolution of motor and non-motor symptoms or the progression rates of different PD subtypes but rather to evaluate the discriminative power of neuroimaging data in distinguishing them. The follow-up data also ensure, with a sufficient level of clinical certainty, the exclusion of patients with atypical Parkinsonism, such as multiple system atrophy.

In conclusion, our study highlights the clinical significance of non-motor symptoms, such as RBD, constipation, and neurogenic orthostatic hypotension, in identifying a potentially more malignant phenotype in PD, particularly in relation to non-motor symptoms. However, presynaptic dopaminergic imaging did not support a clear distinction between the “Body-First” and “Brain-First” subtypes in early PD patients. Specifically, we did not observe the expected greater symmetry of nigrostriatal degeneration in those clinically classified as “Body-First”. These findings suggest that the “Body-First” vs “Brain-First” hypothesis may not be fully supported by imaging at the time of diagnosis, although differences could emerge in the prodromal phase. Notably, “Brain-First” PD appears more closely associated with central dopaminergic dysfunction compared to “Body-First” PD, reinforcing the idea that distinct pathophysiological processes underlie disease progression. Further research in the earlier stages of PD is necessary to refine these findings, ultimately paving the way for tailored approaches to understanding and managing PD subtypes.

## Methods

### Study participants

We retrospectively studied the imaging and clinical data of 158 de novo PD patients who were referred to the Movement Disorders Clinic of the Neurology Unit at Santa Chiara Hospital, Pisa University (Pisa, Italy) between 2016 and 2018. All patients fulfilling the diagnosis of clinically established PD according to Movement Disorder Society (MDS) criteria^52^ were required to have undergone their first evaluation at our Center (T0) within 36 months of the onset of symptoms, and had undergone a 123 ioflupane-fluoropropyl-carbomethoxy-3-beta-4-iodophenyltropane SPECT ([^123^I]-FP-CIT-SPECT) at our Center within two years of their first symptom onset. The 36-month cutoff was selected to ensure inclusion of patients still in the early stages of disease while maintaining a feasible sample size for long-term follow-up. This threshold reflected a pragmatic compromise, based on the clinical referral patterns and recruitment dynamics of our center, and was deemed appropriate for the goals of this retrospective longitudinal study. Other inclusion criteria included a minimum of six years of follow-up observation and at least one annual evaluation using the same clinical scales administered at baseline.

We divided our cohort into possible “Brain-First” (*top–down*) and “Body-First” (*down–top*) PD based on three different non-motor variables recorded during the baseline clinical evaluation. Specifically, we examined the presence of *down–top* features—namely RBD, constipation, and neurogenic orthostatic hypotension—at baseline to better define the different subtypes. Patients were classified as “Body-First” if they exhibited at least one of these down–top features.

PD patients with RBD (RBD^+^) were those diagnosed with video polysomnography-confirmed (vPSG) RBD and who also reported subjective RBD symptoms at least one year before the onset of motor symptoms. At baseline, patients were assessed using the RBD screening questionnaire (RBDSQ)^53^ and underwent structured interviews conducted by experienced movement disorder specialists. These interviews included detailed questions about dream-enactment behaviors, frequency, and their temporal relationship to motor symptom onset, and were conducted both with the patient and, when available, a bed partner or caregiver. v-PSG was not performed systematically in all patients, but only in those with clinical suspicion of RBD based on a combination of RBDSQ scores and interview findings. RBD was confirmed in 40 patients (~25% of the cohort). All v-PSG recordings were evaluated by board-certified sleep medicine specialists, and the diagnosis of RBD was made according to international criteria^54^. Only patients with both a v-PSG-confirmed diagnosis of RBD and reliable historical documentation—via structured interview—that symptoms were present at least one year before motor onset were classified as “Body-First”. Patients lacking this temporal information, even if v-PSG positive, were excluded from the “premotor RBD” group.

Constipation was defined as occurring at least “sometimes” (score ≥ 3) on items 5–7 of the scale for outcomes in Parkinson’s disease for autonomic symptoms (SCOPA-AUT) questionnaire^55^. Additionally, the Rome IV criteria were used to confirm the diagnosis of constipation^56^. Patients were labeled as constipated (C^+^) if their medical history documented the presence of constipation at least one year before the onset of motor symptoms. This dual-definition approach was adopted to increase diagnostic specificity and reduce the inclusion of non-specific or transient cases.

The “cardiovascular domain” (items 14–16) of the SCOPA-AUT was used to identify pre-diagnostic orthostatic symptoms (score ≥ 3). Patients were then screened for orthostatic hypotension based on a documented fall in systolic blood pressure (SBP) of ≥20 mmHg or diastolic blood pressure (DBP) of ≥10 mmHg within 3 min of standing at baseline. In those meetings, this blood pressure drop criterion, additional measurements of supine and standing heart rate (HR) were used to determine the origin of OH: a ΔHR/ΔSBP ratio lower than 0.5 bpm/mmHg after 3 min^57,58^ standing was considered indicative of neurogenic orthostatic hypotension (nOH), allowing us to classify these patients as nOH^+^^19,20^.

If RBD, constipation, or OH appeared after the onset of motor symptoms, patients were classified as the “Brain-First” subtype. We also collected the following baseline clinical variables: (i) MDS-sponsored revision of the Unified Parkinson’s Disease Rating Scale, motor section (MDS-UPDRS-III)^59^ for standardized motor examination; (ii) SCOPA-AUT^55^ for other autonomic symptoms; (iii) Hamilton Depression Scale (HAM-D)^60^. for depression and Hamilton Anxiety Scale (HAM-A)^61^. for anxiety; (iv) ^24^mini-mental state examination (MMSE)^62^ and MDS-UPDRS I, subitems 1.1^59^ for cognitive assessment.

The diagnosis of mild cognitive impairment (PD-MCI) at baseline and follow-up, as well as dementia (PDD) at follow-up, was made according to published criteria^63,64^. In particular, PD-MCI was diagnosed based on the MDS Task Force Level II criteria^63^, through a comprehensive neuropsychological assessment. Each patient underwent evaluation with at least two validated tests for each of the five cognitive domains—attention and working memory, executive function, language, memory, and visuospatial function—allowing for both diagnostic classification and cognitive subtyping. Levodopa equivalent daily dose (LEDD) was calculated according to Jost et al.^65^.

We excluded all patients with atypical signs or symptoms suggesting other causes for Parkinsonism (vascular and iatrogenic Parkinsonism or Parkinson’s plus syndromes) and patients carrying any of the existing PD gene mutations. Patients were also excluded if they had a history of stroke, brain injury, or any other major neurological or psychiatric disease, diabetes, hypertension, neuropathies, heart or kidney failure, current or previous cancer, and/or major surgery on abdominal organs, inflammatory bowel disease, severe intracranial or extracranial artery stenosis/occlusion, or history of peripheral arterial disease.

Additionally, none of the patients were on antidepressant, anticholinergics, antipsychotic medications, or alpha-adrenergic antagonists (e.g., for prostate disorders).

For disease progression, we considered the MDS-UPDRS-III, LEDD, and MMSE scores from baseline and annual assessments. In particular, three main outcome measures of progression were selected for examination and comparison between the different groups:

- Percent change from baseline (T0) to the last assessment (T1) in the MDS-UPDRS motor subscore (during the OFF-state)
- Percent change from the two-year assessment to the last assessment (T1) in LEDD
- Percent change from baseline (T0) to the last assessment (T1) in MMSE

During the follow-up evaluations, UPDRS IV was also recorded to provide information on the development of motor fluctuations and dyskinesias.

#### DAT-SPECT protocol

Images were acquired 3–5 h after intravenous injection of 185 MBq [^123^I]-FP-CIT using a hybrid SPECT/CT scanner (Discovery NM/CT 670, GE Healthcare Technologies, Waukesha, WI, USA). This system includes a dual-head gamma camera with large field-of-view detectors (54 × 40 cm) equipped with 3/8″-thick NaI(Tl) crystals, integrated with a 16-slice BrightSpeed™ Elite CT scanner (GE Healthcare Technologies, Waukesha, WI, USA). SPECT acquisition consisted of 240 projections over a 360° circular orbit with 3° angular steps, producing DICOM files with a voxel size of 2.94 × 2.94 × 2.94 mm. To optimize crystal use and minimize dead space, the field of view was shifted toward the patient’s shoulders (PanY = −20). Low-energy, high-resolution (LEHR) collimators were used. Two energy windows were set: 159 keV (±20%) for ^123^I and 130 keV (±20%) for scatter correction. The acquisition time was approximately 35 min, enabling detection of 1.5 million counts in most studies. CT parameters were fixed at 120 kV and 80 mA, without a scout view for current modulation. The CT scan limits, encompassing the entire brain volume, were determined directly on the scintigraphic image. All studies were visually inspected for motion artefacts; when motion was detected, raw projection data were corrected using the vendor’s standard motion-correction algorithm before reconstruction.

#### DaTQUANT analysis

DAT-SPECT images were analyzed using DaTQUANT® software (G.E. Healthcare), which quantitatively compares patient scans to an embedded vendor-provided normative database of healthy controls. This database was developed and validated by GE Healthcare using multi-center data, including subjects from the Parkinson’s Progression Markers Initiative (PPMI) study^66^. The software enables automated assessment of radiotracer binding within bilateral striatal and substriatal regions, using the occipital cortex as the reference region. Images are automatically reoriented, and predefined VOI templates are applied to ensure anatomical consistency, minimizing interobserver variability; the automatic VOI placement is reported to be >95% accurate, with optional manual adjustment when needed^67^. Representative examples of DaT SPECT images from our cohort, with DaTQUANT-defined VOIs overlaid on the striatum and the occipital reference region, are shown in Supplementary Fig. S1.

The primary outputs from DaTQUANT included in this study were the specific binding ratios (SBRs) for the striatum, caudate, and putamen bilaterally, as well as for the anterior and posterior putamen regions. SBRs were calculated as the difference between the mean counts in the region of interest and the reference region, divided by the mean reference counts. Additional measures included the putamen-to-caudate (P/C) ratios for both hemispheres, and the AIs for the striatum, caudate, and putamen. The AI was calculated using as:

1 $${Asymmetry}={abs}({\rm{Cr}}-{\rm{Cl}})/(({\rm{Cr}}+{\rm{Cl}})/2)$$

where “C” denotes the SBR value for the right (Cr) or left (Cl) hemisphere and “abs” denotes the absolute value, ensuring that the index value is always positive. Higher AI values indicate greater asymmetry between hemispheres. Finally, *Z*-scores were computed for both SBRs and AIs by comparing patient values to the embedded normative database reference values, thereby quantifying deviations from the norm.

It should be noted that no raw control scans from PPMI were manually imported or processed. Instead, DaTQUANT automatically compares patient values to its embedded normative database, which includes healthy controls from multicentre studies such as PPMI. This approach is standard in both clinical and research DaT-SPECT analysis, and our main outcome measures (AIs) are minimally affected by potential inter-scanner variability, as they are calculated within each individual scan.

#### Radiomics analysis

Radiomics features were extracted from DAT-SPECT images using the standard VOIs defined by DaTQUANT. To ensure consistency, images were first normalized to the mean intensity of the reference region. Notably, the numerical outputs from DaTQUANT (e.g., SBRs, P/C ratios, and AIs) were not used as radiomics features. Instead, the VOIs defined by DaTQUANT were applied to the normalized images to ensure anatomical consistency for feature extraction. PyRadiomics was then used to extract 94 radiomics features from six VOIs, with a fixed bin count of 70. These features included 19 first-order statistics, 24 gray-level co-occurrence matrix (GLCM) features, 16 gray-level run length matrix (GLRLM) features, 16 gray-level size zone matrix (GLSZM) features, 5 neighborhood gray-tone difference matrix (NGTDM) features, and 14 gray-level dependence matrix (GLDM) features.

The dataset was analyzed using a logistic regression model with elastic net regularization, following an approach adapted from Francischello et al.^68^. Model performance evaluation and hyperparameter tuning were performed within a nested cross-validation framework to avoid positive bias in performance estimates^30^. The external CV scheme consisted of a Monte Carlo cross-validation with 300 repetitions, each involving a random stratified split of the dataset into training (60%) and testing (40%) subsets to preserve class proportions. Within each training subset, an internal 5-repetition 2-fold CV was used for feature selection and hyperparameter tuning via a grid search procedure. Feature selection was performed using an L1-regularized step, with the intensity of the L1 penalty treated as a tunable parameter. The classifier was an extreme gradient boosting (XGBoost) model, for which the L1/L2 regularization ratio and the overall regularization strength were optimized. Final model performance metrics were computed on the independent test sets from each Monte Carlo iteration and averaged across all 300 repetitions to obtain robust estimates of generalizability.

### Ethics approval and consent to participate

The research was performed in accordance with the Declaration of Helsinki. Ethical approval was obtained from the local ethical committee. Written informed consent was obtained from all participants.

### Statistical analysis

Continuous variables are presented as means ± standard deviations (SD), while nominal data are summarized as absolute numbers and percentages. The normality of continuous variables was assessed using the Shapiro-Wilk test. For group comparisons, Student’s *t*-test was applied to normally distributed continuous variables, while categorical variables were analyzed using the Chi-square test.

The *compareGroups* package (RStudio) was used for subgroup comparisons of the DaTQUANT output and for generating corresponding summary tables. Continuous variables derived from DaTQUANT outputs were summarized as means ± SD.

A multivariate multiple regression approach was employed to assess the simultaneous relationships between multiple dependent variables (SBRs of DaTQUANT output) and a set of independent predictors, including “Brain-First”/“Body-First” subtype, sex, age at baseline, years of disease at baseline, and baseline MDS-UPDRS-III score. This method was selected to account for the inherent correlations among the dependent variables, while evaluating both the combined and individual effects of the predictors.

To explore potential differences in dopaminergic involvement, subgroup analyses were performed separately for “Brain-First” and “Body-First” subtypes. Multivariate regression models were fitted for each subgroup, with SBRs as the response variables and clinical and demographic variables as predictors.

To account for potential collinearity between LEDD and MDS-UPDRS-III in longitudinal comparisons, ANCOVA models were applied in which each variable was analyzed while adjusting for the other as a covariate. Adjusted *p*-values were derived accordingly and used for group comparisons.

Summary statistics, regression coefficients, and *p*-values were reported to highlight significant findings. All statistical tests were two-tailed, and a *p*-value < 0.05 was considered statistically significant. All statistical analyses were conducted using SPSS software (version 29 for MS Windows, Chicago, USA) and RStudio software (Version 2024.12.0 + 467).

## Supplementary information

Supplementary Information

## Data Availability

The datasets generated and/or analyzed during the current study contain sensitive patient information and are not publicly available due to privacy and ethical restrictions. De-identified derived data (e.g., DaTQUANT outputs, radiomics feature matrices, and summary statistics) can be shared upon reasonable request to the corresponding author and following approval by the local ethics committee(s) and execution of a data-sharing agreement. Analyses used standard, publicly available software (DaTQUANT®, PyRadiomics, XGBoost, R, and SPSS). Custom scripts used to preprocess data, extract features, and run the nested cross-validation workflow are available from the corresponding author upon reasonable request.

## Code availability

Analyses used standard, publicly available software (DaTQUANT®, PyRadiomics, XGBoost, R, and SPSS). Custom scripts used to preprocess data, extract features, and run the nested cross-validation workflow are available from the corresponding author upon reasonable request.
