# Supplementary material for: Discriminating between proposed Brain-First and Body-First Parkinson’s disease using conventional and radiomics-enhanced dopamine transporter SPECT image analysis
Source: NPJ Parkinsons Dis. 2025 Nov 20;11:328. doi: 10.1038/s41531-025-01164-z (PMC12635385; doi:10.1038/s41531-025-01164-z)
Supplement: Supplementary file 1 — Supplementary Information [file 41531_2025_1164_MOESM1_ESM.pdf]

**Table S1. Comparison between PD patients with and without RBD at baseline**			
	**RBD⁺ (n.40)**	**RBD⁻ (n.118)**	**p value**
Female/Male, n.	14/26	54/64	0.235
Age at inclusion, y	65.6 ± 8.9	63.7 ± 10.4	0.330
Age at symptom onset, y	64.4 ± 8.9	62.4 ± 10.6	0.288
Disease duration (months)	12.3 ± 10.5	14.7 ± 13.7	0.320
Predominant side of motor symptoms (n, %)			0.135
Right	23 (57.5)	52 (44.1)	
Left	17 (42.5)	61 (51.7)	
Bilateral	0	6 (5.1)	
MDS-UPDRS motor subscore (off-state)	19.5±7.5	18.6±7.6	0.605
MMSE	29.1 ± 1.3	29.2 ± 1.6	0.766
RBDSQ	8.9 ± 1.7	2.9 ± 1.4	**<0.001**
Constipation, n. (%)	7 (17.5)	14 (11.8)	0.275
nOH, n. (%)	3 (7.5)	4 (3.4)	0.371
SCOPA-AUT Urinary domain - (total score ≥1), n. (%)	9 (22.5)	12 (10.1)	**0.047**
HAM-D (total score ≥13), n. (%)	18 (45)	37 (31.3)	0.117
HAM-A (total score ≥10), n. (%)	11 (27.5)	28 (23.7)	0.633
MDS-UPDRS 1.1 (score=1), n. (%)	9 (22.5)	13 (11.0)	0.070
MCI, n. (%)	6 (15.0)	8 (6.8)	0.114

Values are mean ± standard deviation if not otherwise indicated.
Abbreviations: HAM-A, Hamilton Anxiety Scale; HAM-D, Hamilton Depression Scale; MCI, Mild Cognitive Impairment; MDS-UPDRS, MDS-Sponsored Revision of the Unified Parkinson's Disease Rating Scale; nOH, neurogenic orthostatic hypotension; RBD, REM sleep behavior disorder; RBD^+, patients with RBD; RBD^-, patients without RBD; RBDSQ, RBD Screening Questionnaire; SCOPA-AUT, Scale for Outcomes in Parkinson's Disease for Autonomic Symptoms.

Table S2. Comparison between PD patients with and without constipation at baseline

	C^+ (n. 21)	C^- (n. 137)	p value
Female/Male	10/11	58/79	0.649
Age at inclusion, y	68.4 ± 8.2	63.5 ± 10.2	**0.040**
Age at symptom onset, y	67.1 ± 8.2	62.3 ± 10.3	**0.046**
Disease duration (months)	18.0 ± 20.8	13.4 ± 12.3	0.184
Predominant side of motor symptoms (n, %)			0.601
Right	9 (42.8)	64 (46.7)	
Left	11 (52.4)	67 (48.9)	
Bilateral	1 (4.8)	6 (4.4)	
MDS-UPDRS motor subscore	20.1 ± 7.5	18.6 ± 7.6	0.413
MMSE	28.9 ± 1.2	29.2 ± 1.6	0.367
RBDSQ	5.7 ± 1.9	5.0 ± 2.0	0.135
nOH, n. (%)	2 (9.5)	5 (3.6)	0.223
SCOPA-AUT Urinary domain - (total score ≥1), n. (%)	7 (33.3)	14 (10.2)	**0.004**
HAM-D (total score ≥13), n. (%)	10 (47.6)	45 (32.8)	0.186
HAM-A (total score ≥10), n. (%)	6 (28.5)	33 (24.1)	0.602
MDS-UPDRS 1.1 (score=1), n. (%)	5 (23.8)	17 (12.4)	0.160
MCI, n. (%)	4 (19.0)	10 (7.3)	0.078

Values are mean ± standard deviation if not otherwise indicated.

Abbreviations: C^+, patients with constipation; C^-, patients without constipation; HAM-A, Hamilton Anxiety Scale; HAM-D, Hamilton Depression Scale; MCI, Mild Cognitive Impairment; MDS-UPDRS, MDS-Sponsored Revision of the Unified Parkinson's Disease Rating Scale; nOH, neurogenic orthostatic hypotension; RBDSQ, RBD Screening Questionnaire; SCOPA-AUT, Scale for Outcomes in Parkinson's Disease for Autonomic Symptoms.

**Table S3. DaTQUANT output for patients categorized by their RBD^{+} versus RBD^{-} status**			
**Variables**	**RBD^{+} (n. 40)**	**RBD^{-} (n. 118)**	**p value**
Striatum Right SBR	1.20 (0.38)	1.22 (0.34)	0.753
Striatum Left SBR	1.43 (0.40)	1.43 (0.42)	0.942
Putamen Right SBR	1.02 (0.32)	1.03 (0.30)	0.843
Putamen Left SBR	1.22 (0.39)	1.21 (0.37)	0.876
Caudate Right SBR	1.59 (0.57)	1.64 (0.55)	0.623
Caudate Left SBR	1.88 (0.52)	1.91 (0.61)	0.762
Anterior Putamen Right SBR	1.18 (0.36)	1.20 (0.35)	0.759
Anterior Putamen Left SBR	1.42 (0.45)	1.39 (0.42)	0.706
Posterior Putamen Right SBR	0.69 (0.28)	0.68 (0.26)	0.825
Posterior Putamen Left SBR	0.80 (0.29)	0.84 (0.33)	0.530
Putamen to Caudate Right Ratio	0.79 (0.10)	0.79 (0.13)	0.757
Putamen to Caudate Left Ratio	0.78 (0.10)	0.77 (0.12)	0.845
Caudate Asymmetry	0.13 (0.10)	0.12 (0.08)	0.643
Putamen Asymmetry	0.11 (0.08)	0.12 (0.08)	0.582
Striatum Asymmetry	0.12 (0.08)	0.11 (0.07)	0.935
Z-Score Striatum Left SBR	-2.81 (0.84)	-2.93 (0.98)	0.488
Z-Score Striatum Right SBR	-2.95 (1.10)	-3.04 (0.96)	0.671
Z-Score Caudate Left SBR	-2.01 (0.98)	-2.07 (1.23)	0.752
Z-Score Caudate Right SBR	-2.02 (1.31)	-2.06 (1.22)	0.872
Z-Score Putamen Left SBR	-2.95 (0.89)	-3.10 (0.90)	0.383
Z-Score Putamen Right SBR	-3.48 (0.85)	-3.60 (0.84)	0.443
Z-Score Anterior Putamen Left SBR	-2.63 (0.99)	-2.82 (0.93)	0.311
Z-Score Anterior Putamen Right SBR	-3.22 (0.89)	-3.30 (0.87)	0.599
Z-Score Posterior Putamen Left SBR	-3.31 (0.69)	-3.35 (0.88)	0.813
Z-Score Posterior Putamen Right SBR	-3.65 (0.80)	-3.84 (0.87)	0.212
Z-Score Striatum Asymmetry	3.49 (3.41)	3.44 (3.15)	0.933
Z-Score Caudate Asymmetry	2.37 (2.86)	2.11 (2.17)	0.605
Z-Score Putamen Asymmetry	2.90 (2.98)	3.23 (3.04)	0.561
Z-Score Putamen to Caudate Left Ratio	-2.25 (1.69)	-2.30 (1.98)	0.868
Z-Score Putamen to Caudate Right Ratio	-2.03 (1.65)	-2.20 (2.10)	0.613

Data are presented as mean and standard deviation (SD). Abbreviations: RBD^{+}, patients with REM behaviour disorder; RBD^{-}, patients without REM behaviour disorder; SBR, specific binding ratio.

**Table S4. DaTQUANT output for patients categorized by their C^+ versus C^- status**			
**Variables**	**C^+ (n. 21)**	**C^- (n. 137)**	**p value**
Striatum Right SBR	1.19 (0.34)	1.22 (0.36)	0.674
Striatum Left SBR	1.41 (0.43)	1.44 (0.41)	0.831
Putamen Right SBR	0.99 (0.28)	1.03 (0.31)	0.513
Putamen Left SBR	1.19 (0.36)	1.21 (0.38)	0.751
Caudate Right SBR	1.62 (0.50)	1.63 (0.56)	0.927
Caudate Left SBR	1.90 (0.66)	1.90 (0.58)	0.956
Anterior Putamen Right SBR	1.17 (0.35)	1.20 (0.36)	0.694
Anterior Putamen Left SBR	1.38 (0.42)	1.40 (0.43)	0.845
Posterior Putamen Right SBR	0.62 (0.20)	0.69 (0.27)	0.171
Posterior Putamen Left SBR	0.79 (0.25)	0.84 (0.33)	0.503
Putamen to Caudate Right Ratio	0.77 (0.09)	0.79 (0.13)	0.334
Putamen to Caudate Left Ratio	0.77 (0.09)	0.78 (0.12)	0.768
Caudate Asymmetry	0.12 (0.08)	0.13 (0.09)	0.869
Putamen Asymmetry	0.12 (0.09)	0.12 (0.08)	0.934
Striatum Asymmetry	0.12 (0.08)	0.12 (0.08)	0.949
Z-Score Striatum Left SBR	-2.76 (0.78)	-2.92 (0.97)	0.414
Z-Score Striatum Right SBR	-2.83 (1.15)	-3.05 (0.97)	0.426
Z-Score Caudate Left SBR	-1.88 (0.97)	-2.08 (1.20)	0.388
Z-Score Caudate Right SBR	-1.84 (1.49)	-2.08 (1.20)	0.488
Z-Score Putamen Left SBR	-2.88 (0.86)	-3.09 (0.90)	0.295
Z-Score Putamen Right SBR	-3.48 (0.66)	-3.59 (0.87)	0.501
Z-Score Anterior Putamen Left SBR	-2.59 (0.98)	-2.81 (0.94)	0.361
Z-Score Anterior Putamen Right SBR	-3.17 (0.75)	-3.30 (0.89)	0.484
Z-Score Posterior Putamen Left SBR	-3.18 (0.57)	-3.36 (0.87)	0.206
Z-Score Posterior Putamen Right SBR	-3.74 (0.57)	-3.80 (0.89)	0.649
Z-Score Striatum Asymmetry	3.50 (3.22)	3.45 (3.22)	0.948
Z-Score Caudate Asymmetry	2.12 (2.11)	2.18 (2.40)	0.905
Z-Score Putamen Asymmetry	3.08 (3.36)	3.16 (2.97)	0.918
Z-Score Putamen to Caudate Left Ratio	-2.36 (1.55)	-2.28 (1.96)	0.828
Z-Score Putamen to Caudate Right Ratio	-2.44 (1.39)	-2.11 (2.07)	0.356

Data are presented as mean and standard deviation (SD). Abbreviations: C^+, patients with constipation; C^-, patients without constipation; SBR, specific binding ratio.

Table S5. Comparison between PD patients with and without RBD at T1

	**RBD^+ (n.40)**	**RBD^- (n.118)**	**p value**
Age	71.6±9.1	70.6±9.8	0.558
MDS-UPDRS motor subsection	30.9 ± 13.6	29.3 ± 12.5	0.617*
LEDD	576.3 ± 231.2	592.9 ± 274.2	0.656*
MMSE	26.1 ± 5.7	28.0 ± 3.9	**0.029**
RBDSQ	9.5 ± 3.2	4.9 ± 1.7	**< 0.001**
SCOPA-AUT Gastrointestinal domain - Constipation (total score ≥1), n. (%)	16 (40)	42 (49.5)	0.617
SCOPA-AUT Cardiovascular domain - (total score ≥1), n. (%)	6 (15)	19 (22.4)	0.869
SCOPA-AUT Urinary domain - (total score ≥1), n. (%)	31 (77.5)	64 (54.2)	**0.009**
HAM-D (total score ≥13), n. (%)	33 (82)	72 (61)	**0.013**
HAM-A (total score ≥10), n. (%)	25 (62)	54 (46)	0.067
MDS-UPDRS 1.1 (score=1), n. (%)	18 (45.0)	37 (31.4)	0.117
MCI, n. (%)	22 (55.0)	35 (29.7)	**0.004**
Dementia, n. (%)	6 (15.0)	9 (7.6)	0.169

Values are mean ± standard deviation if not otherwise indicated. *p-values for LEDD and MDS-UPDRS motor subscore were obtained via ANCOVA adjusted for MDS-UPDRS and LEDD respectively.
Abbreviations: HAM-A, Hamilton Anxiety Scale; HAM-D, Hamilton Depression Scale; LEDD: Levodopa Equivalent Dose; MCI: Mild Cognitive Impairment; MDS-UPDRS, MDS-Sponsored Revision of the Unified Parkinson's Disease Rating Scale; RBD, REM sleep behavior disorder; RBD^+, patients with RBD; RBD^-, patients without RBD; RBDSQ, RBD Screening Questionnaire; SCOPA-AUT, Scale for Outcomes in Parkinson's Disease for Autonomic Symptoms.

**Table S6. Comparison between PD patients with and without constipation at T1**			
	**C+ (n. 21)**	**C- (n. 137)**	**p value**
Age	74.7 ±8.1	70.3 ±9.7	**0.050**
MDS-UPDRS motor subsection	27.0 ± 6.3	29.3 ± 13.1	0.270*
LEDD	569.5 ± 154.6	598.9 ± 276.8	0.280*
MMSE	27.0 ± 5.2	27.6 ± 4.4	0.361
RBDSQ	6.0 ± 2.1	5.3 ± 1.9	0.123
SCOPA-AUT Cardiovascular domain - (total score ≥1), n. (%)	2 (9.5)	5 (3.6)	0.233
SCOPA-AUT Urinary domain - (total score ≥1), n. (%)	15 (71.4)	70 (51.1)	0.082
HAM-D (total score ≥13), n. (%)	15 (71.4)	90 (65.7)	0.604
HAM-A (total score ≥10), n. (%)	13 (61.9)	66 (48.2)	0.241
MDS-UPDRS 1.1 (score=1), n. (%)	10 (47.6)	45 (32.8)	0.186
MCI, n. (%)	12 (57.1)	45 (32.3)	**0.031**
Dementia, n. (%)	3 (14.3)	12 (8.8)	0.421

Values are mean ± standard deviation if not otherwise indicated. *p-values for LEDD and MDS-UPDRS motor subscore were obtained via ANCOVA adjusted for MDS-UPDRS and LEDD respectively.

Abbreviations: HAM-A, Hamilton Anxiety Scale; HAM-D, Hamilton Depression Scale; LEDD: Levodopa Equivalent Dose; MCI: Mild Cognitive Impairment; MDS-UPDRS, MDS-Sponsored Revision of the Unified Parkinson's Disease Rating Scale; RBD, REM sleep behavior disorder; RBD^+, patients with RBD; RBD^-, patients without RBD; RBDSQ, RBD Screening Questionnaire; SCOPA-AUT, Scale for Outcomes in Parkinson's Disease for Autonomic Symptoms.

Table S7. Prevalence and Severity of Motor and Non-Motor Symptoms at last follow-up (T1) in the entire cohort.

Age	70.86 ±9.62
MDS-UPDRS motor subscore (off-state)	29.7 ± 12.8
LEDD (mg)	588.6 ± 263.1
MMSE	27.6 ± 4.5
RBD, n. (%)	61 (38.6%)
Constipation, n. (%)	59 (37.3%)
nOH, n. (%)	25 (15.8%)
RBDSQ	6.2 ± 2.3
SCOPA-AUT Urinary domain (total score ≥ 1), n. (%)	75 (47.5%)
HAM-D (total score ≥ 13), n. (%)	105 (66.5%)
HAM-A (total score ≥ 10), n. (%)	79 (50 %)
MDS-UPDRS 1.1 (score =1), n (%)	55 (34.8%)
MCI, n (%)	57 (36.1%)
Dementia, n (%)	15 (9.5%)

Values are mean ± standard deviation if not otherwise indicated.
Abbreviations: HAM-A, Hamilton Anxiety Scale; HAM-D, Hamilton Depression Scale; MCI, Mild Cognitive Impairment; MDS-UPDRS, MDS-Sponsored Revision of the Unified Parkinson's Disease Rating Scale; MMSE, Mini Mental State Examination; nOH, neurogenic orthostatic hypotension; RBD, REM sleep behavior disorder; RBDSQ, RBD Screening Questionnaire; SCOPA-AUT, Scale for Outcomes in Parkinson's Disease for Autonomic Symptoms.

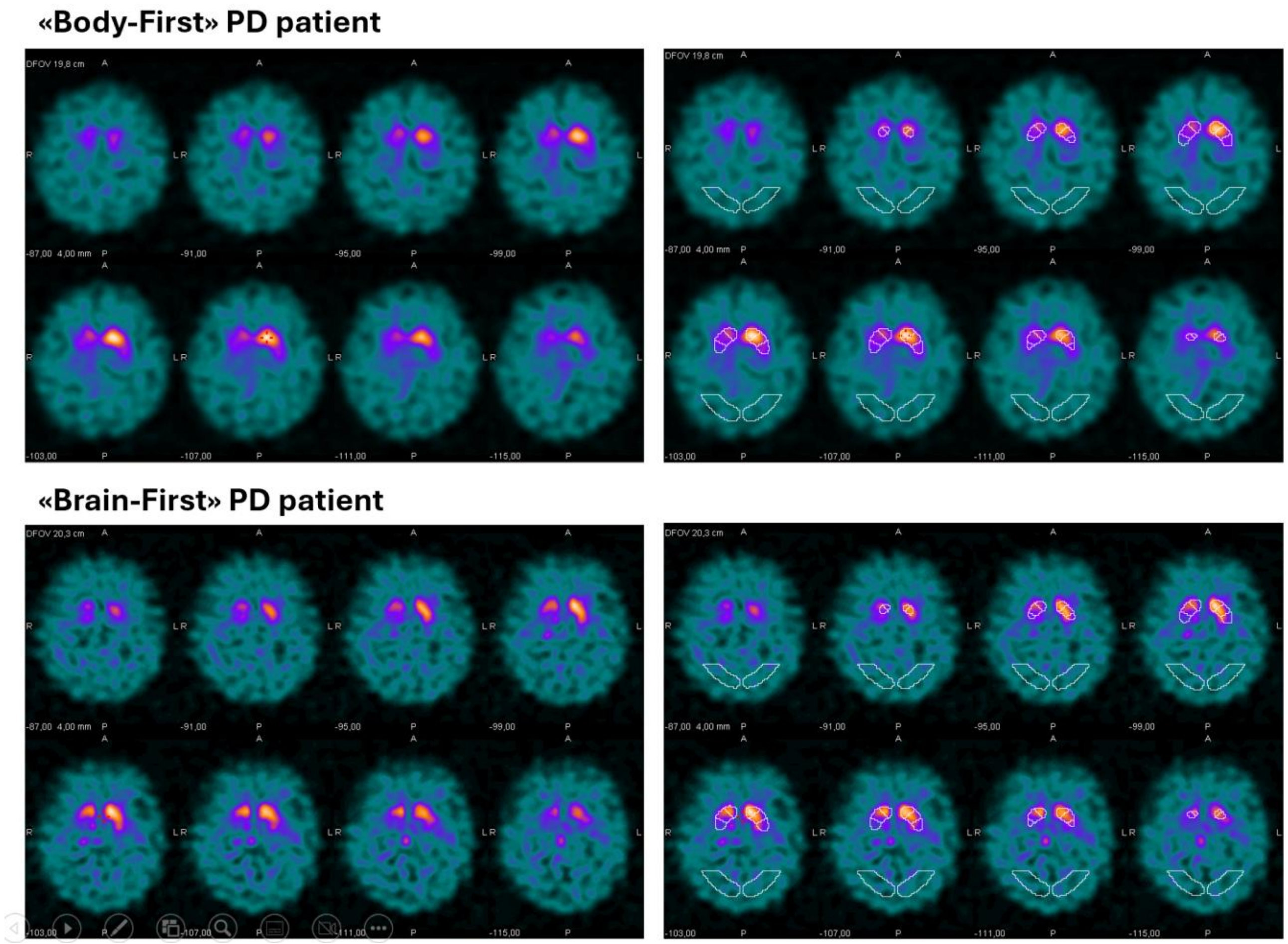

Figure S1. Representative DAT SPECT images from two study participants. *Top panels*: **Body-first PD patient**. *Bottom panels*: **Brain-first PD patient**. For each case, the left images show the original reconstructed SPECT slices, while the right images illustrate the automatically placed volumes of interest (VOIs) for the striatum and occipital reference region as provided by DaTQUANT®. VOI placement was visually inspected and manually corrected only if required, which occurred rarely. These examples illustrate the typical image quality and the robustness of the automated VOI placement in our dataset.
